# Feline leishmaniosis with focus on ocular manifestation: a case report

**DOI:** 10.1186/s13071-023-05741-0

**Published:** 2023-05-12

**Authors:** Ingo Schäfer, Albert Schmidt, Fritz Gräßer, Andrea Schieszler, Heike Aupperle-Lellbach, Gerhard Loesenbeck, Michaela Gentil, Elisabeth Müller, Torsten J. Naucke

**Affiliations:** 1 LABOKLIN GmbH and Co. KG, Bad Kissingen, Germany; 2 Small Animal Practice Dr. Fritz Gräßer, Großostheim, Germany; 3 Tierärztliches Augenzentrum Frankfurt-Kalbach, Frankfurt, Germany

**Keywords:** Vector-borne infection, Vector-borne disease, Ocular leishmaniasis, *Leishmania infantum*

## Abstract

**Background:**

In Europe, feline leishmaniosis is commonly caused by *Leishmania* *infantum*. There is little knowledge regarding pathogenesis, ocular manifestations and long-term follow-ups in cats with leishmaniosis.

**Findings:**

A 6-year-old female, spayed European Shorthair cat was imported from Spain to Germany 2 years prior to its first clinical presentation. The cat showed lethargy, weight loss, ulcerative lesions on the front limbs and high-grade chronic uveitis. The diagnosis of *L. infantum* infection was based on the cytological finding of amastigotes in skin lesions, positive qPCR of EDTA-blood and positive PCR of a cyto-brush sample from the conjunctiva. Supportive findings included positive serology by IFAT, serum protein capillary electrophoresis with peaks in alpha2- and gamma-globulin sections and marked elevation of SAA. Enucleation had to be performed on day 288 on both eyes because of blindness, glaucoma and high-grade uveitis. Histologically, high numbers of *Leishmania* spp. amastigotes were found in histiocytes. IFAT and PCR were positive in the aqueous humor in both eyes, respectively. Feline leukemia virus antigen and feline immunodeficiency virus antibody testings were positive. Hematological and biochemical results revealed mild leukocytosis with lymphocytosis, monocytosis and eosinopenia as well as marked elevation of SAA and hyperglobulinemia. The cat was treated with allopurinol, responded well and was still alive at follow-up on day 288 after first presentation. However, enucleation was necessary because of refractory glaucoma and uveitis.

**Conclusion:**

For the first time, ocular evidence of *Leishmania* IgG antibodies was demonstrated in the aqueous humor of both eyes in cats. There is limited knowledge about the pathogenesis, treatment options and outcomes in cats infected with *L. infantum*. This case report supports the hypothesis that immunosuppression increases the risk of clinical signs of leishmaniasis in cats. Alpha2- and gamma-globulin peaks in serum protein capillary electrophoresis are supportive criteria for the diagnosis of *L. infantum* infection. SAA is valuable for monitoring. Regarding ophthalmology, uveitis and glaucoma may have a poor prognosis.

**Graphical abstract:**

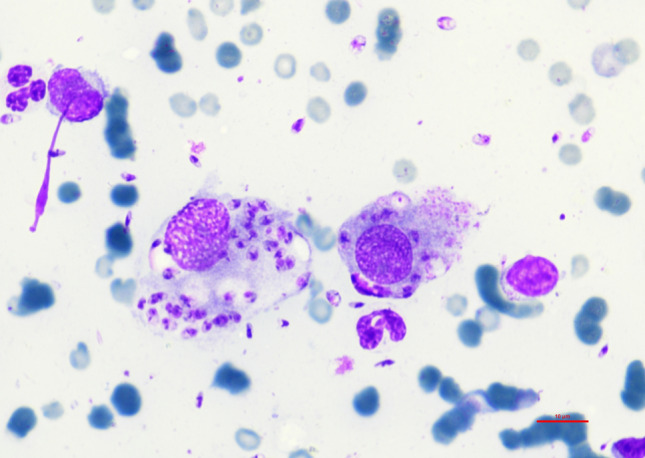

**Supplementary Information:**

The online version contains supplementary material available at 10.1186/s13071-023-05741-0.

## Main text

In Europe, feline leishmaniosis is most often caused by *Leishmania* *infantum* in the Mediterranean area [1–4]. In general, leishmaniosis is considered a globally emerging disease, with phlebotomine sandflies as primary vectors and dogs as the main pathogen reservoir, while cats are accidental hosts in endemic countries [5, 6]. In addition to vector transmission, blood transfusion [2, 7–10], vertical transmission [11], bite wounds [12] and venereal transmission [13] have been suggested as other potential sources for infections in cats and dogs.

The most common clinical manifestations in cats include dermatological signs such as skin and mucocutaneous lesions [4, 14, 15] and ophthalmological signs, i.e. conjunctival, corneal, uveal and retinal lesions [1, 4, 16, 17], as well as non-specific signs like lethargy, anorexia and weight loss [1, 4]. Feline leishmaniosis is most often reported in adult cats with a median age of 7 years [18]. An impaired immune system seems to predispose cats to the development of clinical signs, for example concurrent FIV and/or FeLV infections, neoplasia, immune-mediated diseases and treatment with immunosuppressive drugs such as corticosteroids [4]. Cats with leishmaniosis and clinical signs usually have high levels of parasitemia and hyperglobulinemia, while their antibody levels may be low or high [19].

For diagnosis, direct (PCR, cytology, histology, immunohistochemistry, culture) and indirect detection methods (IFAT, Western blot, direct agglutination test) are available [18]. Information regarding treatment options in cats is minimal and based on individual case reports [15, 20–25]. Treatment of cats with clinical leishmaniosis is still empirically based and off label, using drugs usually administered to dogs [1, 18, 19].

TaqMan^®^ real-time PCR testing was performed at LABOKLIN (Bad Kissingen, Germany); cycle threshold (cq) values < 35 were considered positive. Each PCR run included a negative and a positive control as well as an extraction control in each sample to check for nucleic acid extraction and PCR inhibition (DNA/RNA Process Control Detection Kit, Roche Diagnostics GmbH, Mannheim, Germany). Testing for *Leishmania* spp. by PCR was done using EDTA blood, aqueous humor and a cyto-brush sample collected from the conjunctiva (target: kinetoplast minicircle DNA; primer: 5ʹ—AAC TTT TCT GGT CCT CCG GGT AG—3ʹ, 5ʹ—ACC CCC AGT TTC CCG CC—3ʹ; probe: 5ʹ—FAM—AAA AAT GGG TGC AGA AAT—NFQMGB—3ʹ [26]). Testing for *Hepatozoon* spp. (target: 18S rRNA; primer: 5ʹ—AAC ACG GGA AAA CTC ACC AG—3ʹ, 5ʹ—CCT CAA ACT TCC TCG CGT TA—3ʹ; probe: 5ʹ—FAM—TCA CCC TAT TTA GCA GGT TAA GGT CTC GT—BBQ—3ʹ; own methodology), *Dirofilaria* spp. (target: 5.8S rDNA, primer: 5ʹ—AGT GCG AAT TGC AGA CGC ATT GAG—3ʹ, 5ʹ—AGC GGG TAA TCA CGA CTG AGT TGA- 3ʹ; probe: 5ʹ—FAM—TGA GCA CAA AGA TTT CGA AYG CAC ATT G—BHQ1—3ʹ [27]), *Rickettsia* spp. (target: 23S rRNA; primer: 5ʹ—AGC TTG CTT TTG GAT CAT TTG G- 3ʹ, 5ʹ—TTC CTT GCC TTT TCA TAC ATC TAG T—3ʹ; probe: 5ʹ—FAM—CCT GCT TCT ATT TGT CTT GCA GTA ACA CGC CA—BHQ-1—3ʹ [28]) and *Bartonella henselae* (target: Alr-gcvP IGS, primer: 5ʹ—GAG GGA AAT GAC TCT CTC AGT AAA A—3ʹ, 5ʹ—TGA ACA GGA TGT GGA AGA AGG—3ʹ; probe: 5ʹ—FAM—CAG CCA AAT ATA CGG GCT ATC CAT CAA—BHQ-1—3ʹ [29]) was performed on EDTA-blood respectively. PCR testing for calicivirus (target: ORF1; primer: 5ʹ- GTT GGA TGA ACT ACC CGC CAA TC—3ʹ, 5ʹ—CAT ATG CGG CTC TGA TGG CTT GAA ACT G—3’; probe: 5ʹ- FAM—TCG GTG TTT GAT TTG GCC TG—BHQ-1—3ʹ [30]) was performed on an oral swab.

For serological detection of *Leishmania* spp., ELISA testing (NovaTec VetLine Leishmania ELISA, Immundiagnostica GmbH, > 11 LE positive) and IFA testing (MegaFLUO^®^ LEISH, MegaCor Diagnostik GmbH, Hörbranz, Austria; > 1:64 positive) were used on serum and aqueous humor respectively. For IFA testing of serum at LABOKLIN, antibodies were detected for *Ehrlichia* spp. by MegaFLUO^®^ EHRLICHIA canis (MegaCor Diagnostik GmbH, Hörbranz, Austria; ≥ 1:40 positive) and for *Rickettsia* spp. by RICKETTSIA CONORII IFA SLIDE (Viracell, Granada, Spain; > 1:128 positive) on serum. Testing for FIV was done by NovaTec VetLine Feline Immunodeficiency Virus ELISA and for FeLV antigen by NovaTec VetLine Feline Leukemia Virus Antigen ELISA (NovaTec Immundiagnostica GmbH, Dietzenbach, Germany) on serum at LABOKLIN. Western blot analysis for FIV from serum was done at the Clinical Laboratory, Department for Clinical Diagnostics and Services of the Vetsuisse Faculty (University of Zurich, Switzerland) and was considered positive if two bands with a molecular weight of 15,000 (p15) and 24,000 (p24) Dalton, respectively, were recognizable on the blotting strip.

**Fig. 1:**
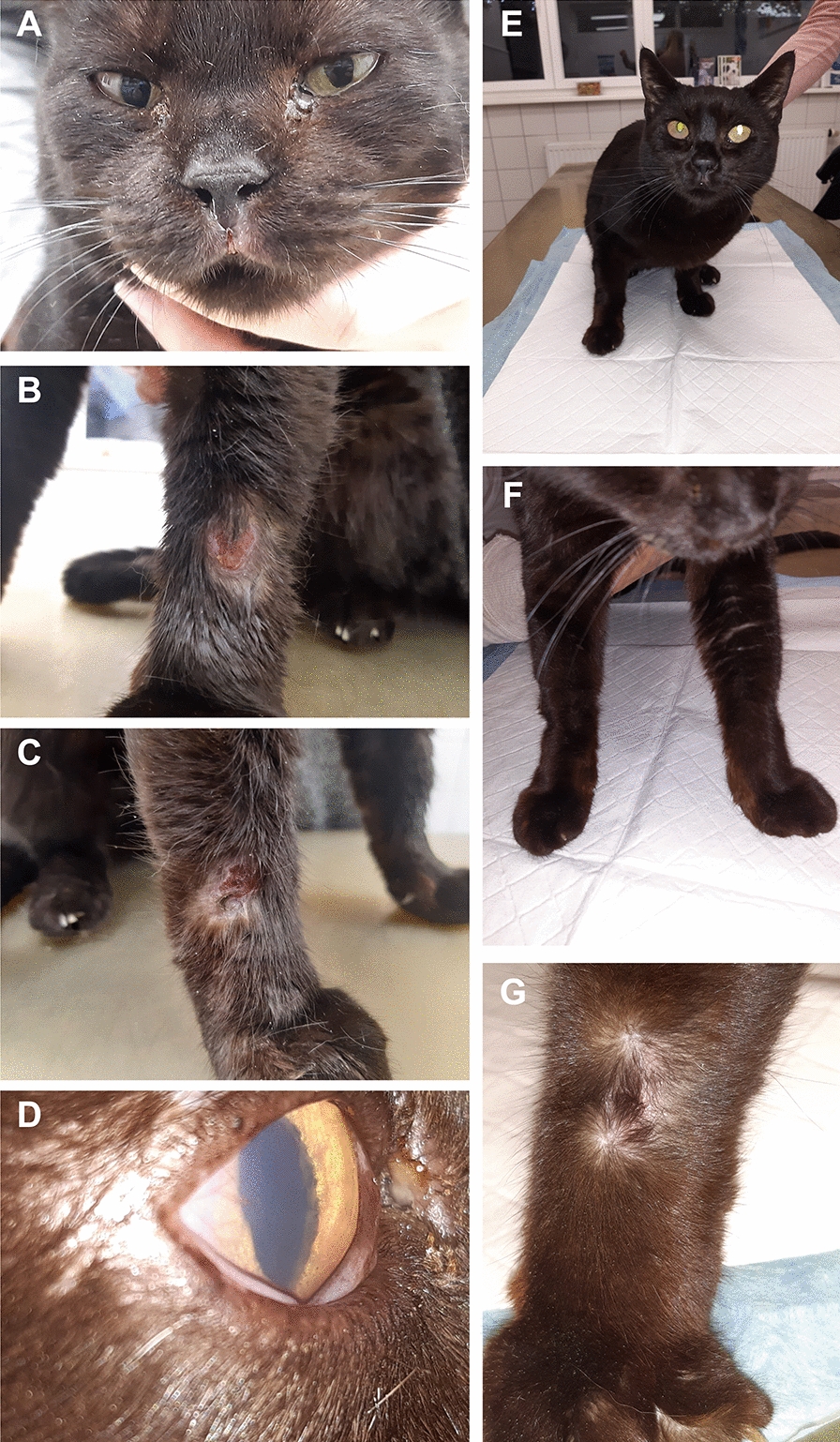
6-year-old female spayed European Shorthair cat imported from Spain to Germany tested positive for *Leishmania* spp. on PCR, antibody ELISA and IFAT. **A** First presentation with nasal stridor, ocular discharge and small ulcerative lesion on the muzzle. **B** Ulcerative lesion on the left carpus at first presentation. **C** Ulcerative lesion on the right carpus at first presentation. **D** Corneal opacity in the right eye at first presentation. **E** Presentation on day 29 after starting treatment with allopurinol. **F** and **G** On day 29 after starting treatment with allopurinol, the ulcerative lesions healed on both front limbs

A 6-year-old female spayed European Shorthair cat was presented to the small animal practice of Dr. Fritz Gräßer (Großostheim, Germany) with lethargy, weight loss (2.8 kg body weight, body condition score 2/9 [31]), ocular discharge (Fig. 1A) and focal ulcerative lesions on the front limbs (Fig. 1B, C). The cat had been imported from Spain to Germany 2 years earlier and was kept indoors in Germany. Initially, there was moderate ulcerative gingivitis and frequent sneezing at first presentation after import. The cat tested positive for calicivirus on PCR, indicating cat flu. One year later, respiratory signs worsened, and a rhinal endoscopy was performed, leading to a diagnosis of chronic rhinitis. The cat was treated with doxycycline (Doxybactin^®^ 50 mg tablets, Dechra, 10 mg/kg once daily orally), meloxicam (Metacam^®^ 0.5 mg/ml suspension for use in cats, Boehringer-Ingelheim, 0.1 mg/kg once daily orally) and inhaled dexamethasone (50 ml dexamethasone 2% with 500 ml sodium chloride) for relief of clinical signs. Three months after starting corticosteroid treatment, the owner noticed ulcerative lesions on both front limbs as well as corneal opacity (Fig. 1A–D). The cat was again taken to the small animal practice of Dr. Fritz Gräßer (Großostheim, Germany). The time of this re-presentation is defined as day 0 in the following article. A general examination revealed nasal stridor and a high-grade ulcerative gingivitis. Scrapes were taken from ulcerative lesions noted on the dorsal aspects of the carpal joints and sent for cytological examination. The remainder of the general examination was unremarkable (i.e. rectal temperature 38.8 °C; pulse frequency 148/min, no lymph adenomegaly).

Ophthalmological examination performed in a specialist center (Tierärztliches Augenzentrum in Frankfurt-Kalbach, Germany) revealed a negative menace response in the right and a positive response in the left eye. The dazzle reflex was positive in both eyes. The pupillary light response was not evident in either eye; dyscoria and posterior synechia were present. Rebound tonometry (Icare^®^ TONOVET, Helsinki, Finland) was used for the measurement of intraocular pressure and revealed 7 mmHg and 4 mmHg for the right and left eye, respectively. In the cornea of the right eye, a central focal erosion with a diameter of 2 mm accompanied by mild diffuse edema was seen using a slit lamp (Kowa SL-15L, Kowa, Japan). They also noted the presence of keratic precipitates especially in the ventral half of the corneae as well as fibrin clots in the anterior chamber of both eyes. The fundus was not visible in the right and unremarkable in the left eye using an indirect ophthalmoscope (Heine omega 500 LED, Heine Optotechnik GmbH & Co. KG, Gilching, Germany). Cytology of a cyto-brush sample using Diff-Quik Romanowsky stain at LABOKLIN revealed moderate inflammation dominated by macrophages. Additionally, low numbers of neutrophils and moderate numbers of extracellular coccoid bacteria were present. There was no cytological evidence for *Leishmania* amastigotes. However, testing of the cyto-brush for *Leishmania* spp. by PCR was positive.

A complete blood count (CBC; ADVIA 2120i, Siemens Healthineers), biochemical profile including SAA (Cobas 8000, Roche), symmetric dimethylarginine (SDMA, manual enzyme-linked immunosorbent assay) and serum protein capillary electrophoresis (Sebia Minicap, Sebia) were carried out. Screening for vector-borne infectious pathogens was performed using a “Feline travel profile” (PCR: *Hepatozoon* spp. and *Dirofilaria* spp.; IFAT from serum: *Ehrlichia* spp., *Leishmania* spp. and *Rickettsia* spp.) with additional testing for *Leishmania* spp. by quantitative PCR testing. Urinalysis (Cobas u601, Roche) was done at LABOKLIN (Bad Kissingen, Germany), including microscopy of urinary sediment and urinary protein-creatinine ratio (UPC, Cobas 8000, Roche).

**Fig. 2:**
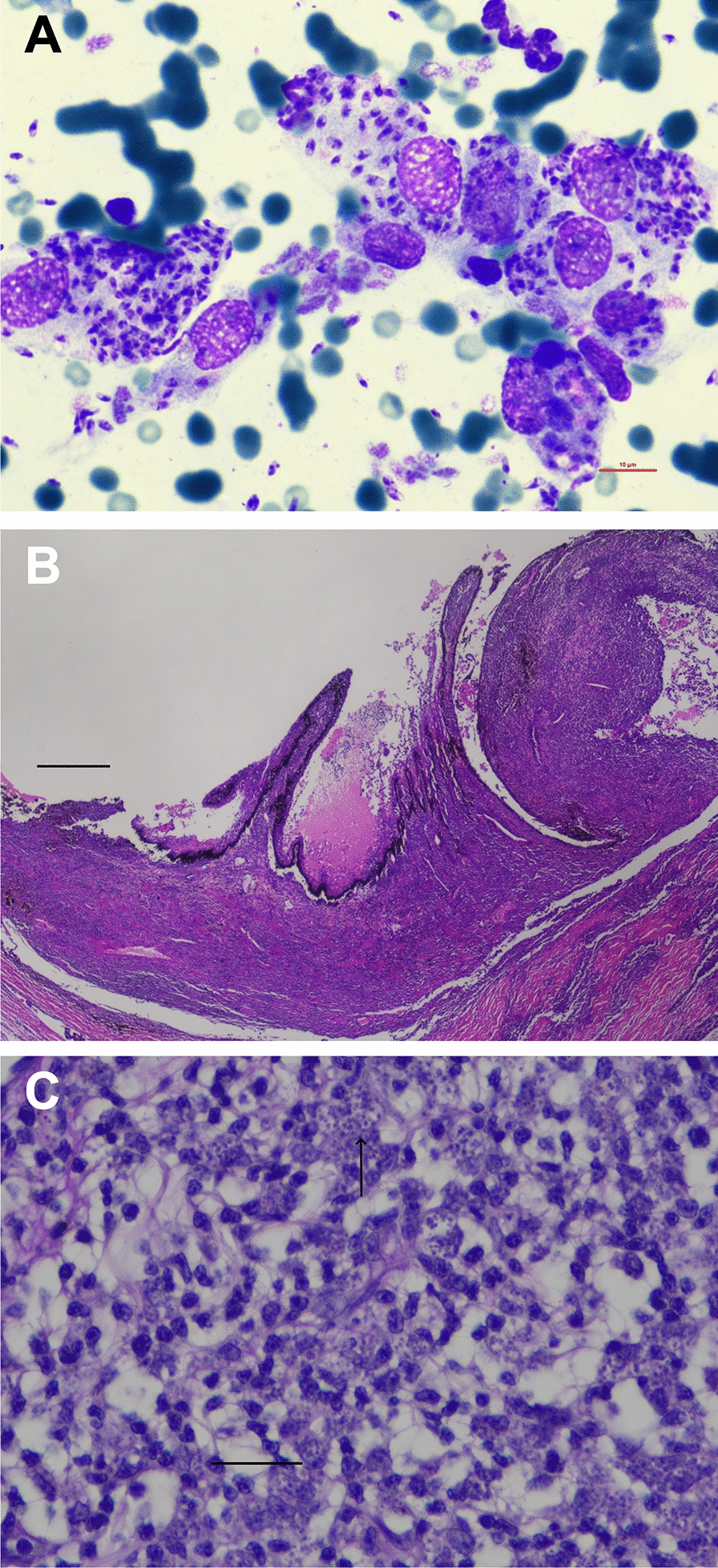
**A** Cytology of ulcerative lesions in a 6-year-old European Shorthair cat imported from Spain to Germany with granulomatous inflammation and high numbers of intra- and extracellular amastigotes of *Leishmania* spp. **B** High-grade lymphohistiocytic iridocyclitis in a 6-year-old cat with feline leishmaniosis, hematoxylin eosin stain, bar 500 µm. **C** Intraocular lymphohistiocytic inflammation with intracellular *Leishmania* spp. amastigotes (black arrows) in a 6-year-old cat with feline leishmaniosis (hematoxylin-eosin staining, according to the standard operation procedures in the LABOKLIN Laboratory, Bad Kissingen, Germany), bar 10 µm

Hematology revealed mild leukocytosis with mild lymphocytosis, mild monocytosis, eosinopenia, mild reticulocytosis and mild thrombocytopenia (Table 1). The biochemistry profile showed mild hyperglobulinemia, marked elevation of SAA, mild elevations in triglycerides and cholesterol, mild hyperkalemia and decreased iron concentration (Table 1). Serum protein electrophoresis showed polyclonal peaks in the alpha2 and gamma sections (Additional file 1). Testing for FeLV antigen, FIV antibodies and FIV Western blot was positive. Within the “feline travel profile,” IFAT for *L. infantum* was positive with a titer of 1:4096 and for *Rickettsia* with a titer of 1:256; testing for all other pathogens included in the panel was negative. A quantitative *Leishmania* spp. PCR was positive with 786 *Leishmania* organisms/ml blood. Cytology using Diff-Quik Romanowsky stain revealed high numbers of intra- and extracellular amastigotes (Fig. 2A). PCR testing for *Rickettsia* spp. and *Bartonella henselae* was negative.

**Table 1:** Hematological and biochemical parameters in a 6-year-old female-spayed European Shorthair cat imported from Spain to Germany infected with *Leishmania infantum*

Parameter	RI	Day 0	Day 48	Day 66	Day 96	Day 117	Day 133	Day 196	Day 288
Hematology									
RBCs (× 10^12^/l)	5.0–10.0	5.82	5.97	6.07	6.85	–	7.74	8.08	7.94
Hemoglobin (g/l)	90–150	96	**83**	**80**	**79**	–	**85**	**86**	**85**
Hematocrit (l/l)	0.3–0.44	0.32	**0.26**	**0.26**	**0.27**	–	0.30	**0.28**	0.3
Reticulocytes (× 10^9^/l)	< 60.0	**64.6**	47.3	**77.9**	**86.3**	–	51.9	38	40.5
CHr (pg)	> 11.5	16.9	16.4	15.1	13.0	–	14.0	13.3	14.7
WBCs (× 10^12^/l)	6.0–11.0	**13.7**	**11.3**	7.9	**12.8**	–	**11.7**	10.8	**12.4**
Segmented (× 10^9^/l)	3.0–11.0	7.1	6.6	3.0	6.7	–	7.1	6.2	7.2
Bands (× 10^9^/l)	< 0.6	0.0	0.0	0.0	0.0	–	0.0	0.0	0.0
Lymphocytes (× 10^9^/l)	1.0–4.0	**5.9**	3.6	4.0	**4.2**	–	4.0	3.5	4.0
Eosinophils (× 10^9^/l)	0.04–0.6	**0.0**	0.1	0.6	0.1	–	**0.0**	0.1	**0.0**
Monocytes (× 10^9^/l)	0.04–0.5	**0.7**	**0.9**	0.3	**1.7**	–	**0.6**	**1.1**	**1.2**
Hypochromasia	Negative	Neg.	Neg.	**Pos.**	Neg.	–	Neg.	Neg.	Neg.
Anisocytosis	Negative	Neg.	Neg.	Neg.	Neg.	–	Neg.	Neg.	Neg.
Platelets (× 10^9^/l)	180–550	**175**	337	391	189	–	311	240	257
Biochemistry									
Alpha-Amylase (U/l)	< 1850.0	1291.0	1265.0	1211.0	1198.0	1311.0	–	1374.0	1384.0
DGGR-lipase (U/l)	< 26.0	18.3	20.7	25.0	**27.2**	**32.0**	–	24.5	16.4
Glucose (mmol/l)	3.1–6.9	5.3	4.4	4.6	4.2	5.1	–	5.8	5.3
Fructosamine (µmol/l)	< 340.0	212.0	193.8	200.2	212.9	196.6	–	246.6	212.9
Triglycerides (mmol/l)	< 1.14	**1.2**	0.87	1.05	0.42	**1.55**	–	0.53	0.63
Cholesterol (mmol/l)	1.8–3.9	**5.1**	**4.9**	**4.6**	**4.7**	**4.2**	–	**4.4**	3.5
Bilirubin (µmol/l)	< 3.4	1.1	0.5	2.2	2.3	1.8	–	2.1	**3.6**
ALP (U/l)	< 140.0	16.0	16.0	16.0	17.0	29.0	–	37.0	38.0
GLDH (U/l)	< 6.0	1.8	2.0	**7.0**	4.2	4.6	–	**20.7**	2.5
G-GT (U/l)	< 5.0	< 0.1	< 0.1	< 0.1	< 0.1	< 0.1	–	< 0.1	0.1
ALT (U/l)	< 70.0	18.2	24.3	44.5	32.7	59.3	–	**230.5**	**99.6**
AST (U/l)	< 30.0	20.9	12.4	20.7	16.4	26.6	–	**61.6**	43.3
Creatin kinase (U/l)	< 130.0	38.0	18.0	22.0	30.0	55.0	–	124.0	70.0
Total protein (g/l)	57.0–94.0	85.9	89.9	**97.2**	**101.2**	**100.7**	–	**103.7**	**98.8**
Albumin (g/l)	26.0–56.0	28.9	28.0	26.0	27.1	29.2	–	38.8	30.6
Globulin (g/l)	< 55.0	**57.0**	**61.9**	**71.2**	**74.1**	**71.5**	–	**64.9**	**68.2**
Urea (mmol/l)	5.0–11.3	5.4	6.5	7.1	7.4	8.0	–	8.3	6.5
Creatinine (µmol/l)	< 168.0	92.0	106.0	80.0	94.0	104.0	–	124.0	117.0
Phosphorus (mmol/l)	0.8–1.9	1.9	1.6	1.3	1.5	1.4	–	1.2	1.5
SDMA (µmol/l)	< 0.75	–	–	0.62	0.57	0.47	–	0.56	0.46
Magnesium (mmol/l)	0.6–1.3	0.9	0.9	0.8	0.9	0.9	–	0.9	0.8
Calcium (mmol/l)	2.3–3.0	2.7	2.4	2.3	2.3	2.4	–	2.5	2.3
Sodium (mmol/l)	145.0–158.0	155.0	150.0	148.0	147.0	150.0	–	152	151
Potassium (mmol/l)	3.0–4.8	**5.0**	4.5	4.2	4.7	4.4	–	4.3	4.1
Iron (µmol/l)	8–31	**7.9**	**7.5**	**5.3**	**6.0**	**5.7**	–	**6.5**	**5.5**
Serum amyloid A (µg/l)	< 6.7	**77.4**	**35.2**	**21.4**	**15.2**	**13.6**	–	5.9	**12.0**
Pathogen detection methods									
* Leishmania* IFAT	≤ 1:64	**1:4096**	**1:512**	**1:1024**	**1:1024**	**1:512**	**1:256**	**1:256**	**1:256**
* Leishmania* ELISA	< 11 LE	–	–	–	–	**23.7**	–	**14.7**	**12.9**
* Leishmania* qPCR (/ml)	–	**786** **(pos)**	**191** **(pos)**	**1** **(pos)**	**238** **(pos)**	**94** **(pos)**	–	**11** **(pos)**	**49** **(pos)**

*RI* reference interval, *RBCs* red blood cells, *CHr* reticulocyte hemoglobin content, *WBCs* white blood cells, *Segmented* segmented neutrophilic granulocytes, *Bands* banded neutrophilic granulocytes, *ALP* alkaline phosphatase, *GLDH* glutamate dehydrogenase, *G-GT* gamma-glutamyl transferase, *ALT* alanine transaminase, *AST* aspartate aminotransferase, *SDMA* symmetric dimethylarginine, *ELISA * antibody enzyme-linked immunosorbent assay, *IFAT* immunofluorescence antibody test, *qPCR* quantitative polymerase chain reaction

parameters outside the reference intervals of the commercial laboratory LABOKLIN

Due to the presence of ophthalmological manifestations, Ketorolac-Trometamol and, after healing of the corneal erosion, prednisolone acetate were additionally prescribed on day 75 (Table 2). A qPCR on day 96 revealed an increase of *Leishmania* in the blood to 238 per milliliter, against a background of mild leukocytosis with lymphocytosis and monocytosis, potentially indicating an inflammation due to the immunosuppressive medication. Consequently, the allopurinol dose was doubled to 25 mg BID PO, after which the number of detectable *Leishmania* decreased to 11 *Leishmania*/ml blood on day 196, with SAA within the reference range. On day 288, the cat was presented because glaucoma and enucleation had to be performed on both eyes. SAA showed a mild elevation and 49 leishmania/ml blood were detected in quantitative PCR testing (Table 2).

**Table 2:** Results of qPCR, IFA-testing, hematological analysis and SAA evaluation as well as treatment in a 6-year-old female spayed European Shorthair cat imported from Spain to Germany infected with *Leishmania infantum* from day 0 to day 288

Day	*Leishmania* qPCR^A^ (/ml blood)	*Leishmania* spp. IFAT^B^ ≤ 1:32 negative	Serum amyloid A^C^ < 6.7 µg/l	Hematology^D^	Treatment
0	768	1:4096	77.5	Mild reticulocytosis; mild leukocytosis with lymphocytosis, monocytosis and eosinopenia; mild thrombocytopenia	Allopurinol 12.5 mg BID p.o.^1^ Meloxicam 0.05 mg/kg SID p.o.^2^
48	191	1:512	35.2	Mild non-regenerative anemia; mild leukocytosis with monocytosis	Allopurinol 12.5 mg BID p.o.^1^ Meloxicam 0.05 mg/kg SID p.o.^2^
66	1	1:1024	21.4	Mild regenerative anemia	Allopurinol 12.5 mg BID p.o.^1^ Meloxicam 0.05 mg/kg SID p.o.^2^
75	–	–	–	–	Allopurinol 12.5 mg BID p.o.^1^ Meloxicam 0.05 mg/kg SID p.o.^2^ Prednisolone acetate^3^ after healing of the corneal erosion) and Ketorolac-Trometamol^4^ 1 drop BID in each eye
96	238	1:1024	15.2	Mild regenerative anemia; mild leukocytosis with lymphocytosis and monocytosis	Allopurinol 25 mg BID p.o.^1^ Meloxicam 0.05 mg/kg SID p.o.^2^ Prednisolone acetate^3^, Ketorolac-Trometamol^4^ and Nepafenac^5^ 1 drop BID in each eye
117	94	1:512	13.6	–	Allopurinol 25 mg BID p.o.^1^ Meloxicam 0.05 mg/kg SID p.o.^2^
133	–	1:256	–	Mild leukocytosis with monocytosis and eosinopenia	Allopurinol 25 mg BID p.o.^1^ Meloxicam 0.05 mg/kg SID p.o.^2^ Ophthalmological treatment protocol^E^
196	11	1:256	5.9	Mild non-regenerative anemia, mild monocytosis	Allopurinol 25 mg BID p.o.^1^ Meloxicam 0.05 mg/kg SID p.o.^2^ Ophthalmological treatment protocol^E^
288	49	1:256	12.0	Mild leukocytosis with monocytosis and eosinopenia	Allopurinol 25 mg BID p.o.^1^ Meloxicam 0.05 mg/kg SID p.o.^2^ Enucleation due to uveitis, glaucoma, and blindness

*IFAT* immunofluorescence antibody test, *qPCR* quantitative polymerase chain reaction

^A^quantitative TaqMan real-time PCR (target: kinetoplast minicircle DNA)

^B^MegaFLUO^®^ LEISH, MegaCor Diagnostik GmbH, Hörbranz, Austria; > 1:64 positive

^C^Cobas 8000, Roche

^D^ADVIA 2120i, Siemens Healthineers

^E^Right eye: chloramphenicol 1 drop TID (Cefenicol^®^ CA 5 mg/ml, CP-Pharma, Burgdorf, Germany), ketorolac trometamol^4^ 1 drop SID, Sodiumhyaluronat and dexpanthenol 1 drop SID (Hyalopanthen^®^ for ocular use, CP-Pharma, Burgdorf, Germany); left eye: nepafenac^5^, Pred forte AT, sodiumhyaluronat and dexpanthenol 1 drop SID (Hyalopanthen^®^ for ocular use, CP-Pharma, Burgdorf, Germany), tropicamid 1 drop BID (Mydriaticum Stulln^®^ UD for ocular use, Pharma Stulln GmbH, Stulln, Germany)

^1^Allopurinol-ratiopharm^®^ 100 mg tablets, ratiopharm, Ulm, Germany

^2^Metacam^®^ 0.5 mg/ml oral suspension, Boehringer Ingelheim Vetmedica GmbH, Ingelheim, Germany

^3^Inflanefran^®^ forte 10 mg/ml for ocular use, AbbVie Deutschland GmbH & Co. KG, Ludwigshafen am Rhein, Germany

^4^Acular^®^ 5 mg/ml for ocular use, AbbVie Deutschland GmbH & Co. KG, Ludwigshafen am Rhein, Germany

^5^Nevanac^®^ 1 mg/ml for ocular use, NOVARTIS Pharma GmbH, Nürnberg, Germany

Following enucleation, histopathology of both eyes was performed at LABOKLIN (Bad Kissingen, Germany). Both eyes were sent fixed in 10% neutral buffered formalin. Macroscopically, the cornea was cloudy. The aqueous humor was milky, the vitreous of the eye was not completely transparent, and the retina was detached to a large extent in both eyes. Representative sections were routinely embedded in paraffin wax, and slides were stained by hematoxylin and eosin. Microscopically, there was severe destruction of the inner organs of the eyes accompanied by a severe infiltration of predominantly lymphocytes, histiocytes and, to a lesser extent, neutrophils (Fig. 2B). There was a severe panuveitis (iridocyclitis with infiltration of the cornea, chorioretinitis with extensive detachment of the retina). Many *Leishmania* spp. amastigotes were multifocally detected in the histiocytes (Fig. 2C). The aqueous humor of both eyes was tested by *Leishmania* spp. qualitative PCR (positive, cq in both eyes < 15), *Leishmania* spp. IFAT (right eye: 1:1024, left eye: 1:512) and *Leishmania* spp. ELISA (right eye: 15.1 LE, left eye: 15.3 LE). Besides a mixed inflammation in cytology, no *Leishmania* spp. amastigotes were detected in native and cytocentrifugated slides stained with Diff-Quik Romanowsky stain respectively.

A diagnosis of feline leishmaniosis was made based on the molecular detection of *Leishmania* spp. and supported by serological, cytological and histological detection of the pathogen. Additionally, there were a chronic uveitis and synechia, rhinitis and ulcerative skin lesions.

Urinalysis was performed on day 66 after first presentation and showed a specific gravity of 1030 g/l, pH of 8.0 and UPC of 0.1. There was mild proteinuria (+); all other parameters were unremarkable.

Allopurinol (12.5 mg twice daily orally) application was prescribed following the diagnosis of feline leishmaniosis. The cat was in good condition overall on both day 48 and 66 and was gaining weight (3.0 kg at day 48; 3.3 kg at day 66). Its gingivitis improved to mild to moderate severity, and the ulcerative lesions initially observed were minimal at day 48 (Fig. 1E–G) and no longer visible at day 66. Hematological abnormalities on day 0 to day 288 are listed in Table 2. Regarding biochemistry, mild hyperglobulinemia and low iron were still present on day 48, while SAA was only mildly elevated. On day 66, mild hyperproteinemia with moderate hyperglobulinemia, mild elevation of glutamate dehydrogenase (GLDH), SAA and cholesterol as well as decreased iron concentration were detected (Table 1).

The *Leishmania* spp. IFAT titer was still positive but decreased to 1:512 and 1:1024 on day 48 and 66, respectively. A quantitative *Leishmania* spp. PCR showed 191 *Leishmania*/ml blood on day 48 and 1 *Leishmania*/ml blood at day 66 (Table 1). In addition to IFA testing, a *Leishmania* spp. ELISA was performed using the serum from day 96 onwards (day 96: IFAT 1:512, ELISA 23.7 LE; day 196: IFAT 1:256, ELISA 14.7 LE; day 288: IFAT 1:256, ELISA 12.9 LE).

To the best of the authors’ knowledge, this is the first case report demonstrating *Leishmania* IgG antibodies in the aqueous humor in cats. In wild rabbits from Greece, antibody detection in aqueous humor revealed 100% specificity but decreased sensitivity compared to serum [32]. This indicated the value of aqueous humor in epidemiological studies to confirm exposure at the population level but was interpreted as having little diagnostic value at the individual level. Antibodies in the aqueous humor could be detected because of an increased permeability of the blood aqueous barrier or the local antibody production, especially caused by microorganisms involved in the pathogenesis of uveitis [33, 34]. In dogs with uveitis and *Leishmania* IgG antibody detection in aqueous humor, higher IgG levels in ocular samples were detected compared to the level of antibody in the serum or even in the absence of *Leishmania* IgG antibodies in serum samples [35, 36].

One case report described improvement of ocular signs after 6 months of treatment with allopurinol (10 mg/kg twice daily orally) in *L. infantum* infection in a cat from Brazil [25]. A mixed inflammation was diagnosed by cytology [25], similar to the cat described in this case report. In another case report, ocular improvement was seen 2 months after starting allopurinol therapy in combination with prednisolone, dorzolamide and timolol [21]. To the best of the authors' knowledge, no study has evaluated the effect of allopurinol on intraocular inflammation due to an infection with *Leishmania* spp., and it remains unclear why ophthalmological signs worsened in our cat and enucleation became necessary. However, ocular manifestation of feline leishmaniosis is considered rare, especially compared to dogs. In canine leishmaniosis, it is well known that ocular manifestations are frequent and complications in affected tissues may lead to blindness. In 53 dogs infected with *L. infantum* presenting with ocular and periocular lesions, main ophthalmological findings were keratoconjunctivitis (72%), hyperplasia of conjunctival lymph follicles (55%), blepharitis (51%) and uveitis (21%) [37]. Interestingly, ocular evidence of *L. infantum* IgG antibodies was seen in 74% of infected dogs [37], which was also demonstrated in our cat. High antibody levels were especially detected in dogs with uveitis, as it was also demonstrated in our cat with 1:512 and 1:1024 in IFA testing in the aqueous humor in both eyes, respectively.

Reports of feline leishmaniosis most often concern adult cats (median 7 years) from endemic countries, especially in the Mediterranean area [18, 19], fitting well with our 6-year-old cat imported from Spain which also tested positive for FeLV and FIV. Clinical leishmaniosis in cats is reportedly associated with an impaired immune response, which may be caused by infections such as FeLV or FIV or by immunosuppressive treatment [2, 4, 7, 38–41]. Both the beginning of clinical signs (i.e. day 0) and relapse on day 96 were associated with the local administration of immunosuppressive drugs (Table 2).

This cat was presented with dermatological and ophthalmological signs consistent with feline leishmaniosis. Skin or mucocutaneous lesions are the most common manifestations of feline leishmaniosis, followed by nonspecific clinical signs such as lethargy, anorexia and weight loss [4], in accordance with the findings in this case. The cat’s ulcerative lesions and anterior uveitis may have occurred secondary to the application of dexamethasone. Proliferative and ulcerative chronic inflammation of the oral cavity is also associated with feline leishmaniosis [4]. The gingivitis in this cat may have been secondary to its leishmaniosis, which may also explain the improvement in it after starting allopurinol treatment. While rare, chronic upper respiratory tract diseases with amastigotes in nasal discharge have been reported [16].

In histopathology, diffuse granulomatous inflammation in combination with high numbers of *Leishmania* amastigotes were detected in 5/15 cats with feline leishmaniosis [42]. In general, lesions of feline leishmaniosis are usually accompanied by a high parasite burden suggesting cats as an important reservoir for leishmaniosis [42]. In two of the five cats, panuveitis was diagnosed in histopathology [42]. Another case report revealed a locally extensive area of dermis in the right pinna of a cat with feline leishmaniosis expanded by many epithelioid macrophages containing protozoal amastigotes surrounded by a mixed inflammatory cell infiltrate [43]. The described histopathological findings are consistent with the cat reported in this article.

Diagnosis of feline leishmaniosis is generally based on molecular, serological, cytological or histological findings [1]. In one previous study, 50% of cases were diagnosed by cytology [4]. Cytology is a rapid, inexpensive and non-invasive diagnostic method for leishmaniosis, especially in cats, but it should be confirmed by serology and PCR as a combination of tests is recommended for diagnosis [4].

Non-regenerative anemia is considered one of the most common hematological abnormalities in feline leishmaniasis [4]. In this case, it was noted on day 48. Hyperglobulinemia without hyperproteinemia has been frequently reported [4], and our findings are consistent with this (Table 1). The main alterations in the proteinogram of cats with leishmaniosis appear to be an increase in the alpha-2 fraction and polyclonal gammopathy [44]. It has been suggested that these abnormalities could be indicative of acute infection [44], which would be in accordance with our findings. There are no reports about SAA as a potential marker for successful treatment, like C-reactive protein would be in dogs. SAA has been proposed as a useful predictive indicator of prognosis regardless of diagnosis, but no cats with leishmaniosis were included in the associated study [45]. In our cat, the SAA concentration decreased with improvement of clinical signs and reduction of *Leishmania* in quantitative PCR, indicating this marker may be of some potential benefit.

To the best of the authors’ knowledge, the treatment of clinical feline leishmaniosis is still based on individual case reports of off-label use of drugs known to be effective in dogs [1, 15, 18–25]. In a literature review, different treatment protocols including monotherapy with allopurinol (5–50 mg/kg once or twice daily orally), meglumine antimonate using different protocols, miltefosine (2 mg/kg once daily orally for 30 days), fluconazole (5 mg/kg once daily orally for two months) and itraconazole (50 mg/cat once daily orally for two months) were described [19]. Additionally, meglumine antimonate (50 mg/kg once daily subcutaneously for 30 days) in combination with allopurinol (10 mg/kg once to twice daily orally), meglumine antimonate (50 mg/kg once daily subcutaneously for 30 days) in combination with ketoconazole (10 mg/kg once daily orally) given for three cycles of 4-week duration, allopurinol (10 mg/kg twice daily orally) and domperidone (0.5 mg/kg once daily orally in two courses of 28 days), and metronidazole (25 mg/kg once daily orally) with spiramycin (150,000 UI/kg once daily orally) for 35 days were published [19]. Because in the majority of the published case reports allopurinol was applied as a monotherapy with a clinical improvement in 21 of 29 cats (72%) [19], we decided on a monotherapy with allopurinol, keeping in mind that relapses were reported. The outcome of allopurinol therapy in cats can vary from no response to a clinical cure with recommended dosage of 10–30 mg/kg or 100 mg/cat orally once or twice daily for long-term use [18]. Increased liver enzymes, acute kidney injury, coprostasis and toxidermia are potential side effects of allopurinol in cats [18, 19] but none of these effects have so far been observed in our cat. This may be due to the relatively low allopurinol dose (12.5 mg BID from day 0 to day 96 and 25 mg BID from day 96 onwards, Table 2). The decision was made not to use miltefosine or meglumine antimoniate because of the risk of Heinz body hemolytic anemia [46] and the need for subcutaneous application, respectively.

Monitoring of therapy includes hematological and biochemical examinations, serological testing and quantitative PCR testing. Cats with leishmaniosis usually show high antibody levels [15, 17, 21, 47], which frequently decrease with successful treatment [15, 20, 21]. This was also demonstrated in our cat.

To the best of the authors’ knowledge, this is the first case report indicating ocular evidence of *L. infantum* IgG antibodies. However, this may be more of epidemiological interest. Feline leishmaniasis should be considered as a potential differential diagnosis in cats with stays abroad in countries endemic for *L. infantum*, especially if dermatological and/or ophthalmological signs are present. Further studies are necessary to gain knowledge about therapeutic options and monitoring of therapy as well as about potential ocular production and evidence of IgG antibodies in cats infected with *L. infantum*.

## Supplementary Information

**Additional file 1: **Serum protein capillary electophoresis in a 6-year-old European Shorthair cat infected with *Leishmania infanum* with polyclonal peaks in the alpha 2 (red circle) and gamma section (blue circle).

## Data Availability

All data generated or analyzed during this study are included in this published article.

## Abbreviations

ELISA: Antibody enzyme-linked immunosorbent assay
DNA: Deoxyribonucleic acid
EDTA: Ethylenediaminetetraacetic acid
FeLV: Feline leukemia virus
FIV: Feline immunodeficiency virus
IFAT: Indirect immunofluorescence test
IgG: Immunoglobulin G
LE: Laboratory specific units
PCR: Polymerase chain reaction
qPCR: Quantitative polymerase chain reaction
rDNA: Ribosomal deoxyribonucleic acid
rRNA: Ribosomal ribonucleic acid
SAA: Serum amyloid A
