# Supplementary figures and images for: Feline leishmaniosis with focus on ocular manifestation: a case report
Source: Parasit Vectors. 2023 May 12;16:161. doi: 10.1186/s13071-023-05741-0 (PMC10176290; doi:10.1186/s13071-023-05741-0)

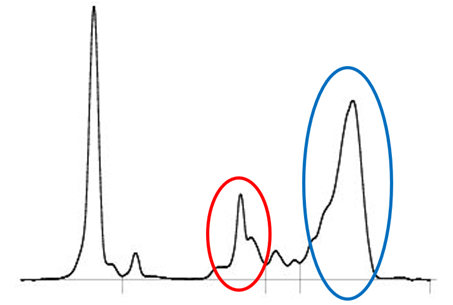

Supplement: Supplementary file 1 — Additional file 1: Serum protein capillary electophoresis in a 6-year-old European Shorthair cat infected with Leishmania infanum with polyclonal peaks in the alpha 2 (red circle) and gamma section (blue circle). [file 13071_2023_5741_MOESM1_ESM.png]
